# Epidemiology and Risk Factors of Irritable Bowel Syndrome in the Saudi Population: A Systematic Review

**DOI:** 10.7759/cureus.79974

**Published:** 2025-03-03

**Authors:** Mansour K Almadi, Mohammed S Sabr, Mostafa Kofi, Talal Alaboodi, Thamer A Al Sayari

**Affiliations:** 1 Family and Community Medicine, Prince Sultan Military Medical City, Riyadh, SAU

**Keywords:** epidemiology, gastrointestinal disorders, ibs, irritable bowel syndrome, prevalence, public health, risk factors, saudi arabia, systematic review

## Abstract

This systematic review examines the available literature on the epidemiology and risk factors of irritable bowel syndrome (IBS) in the Saudi population. A comprehensive electronic search of PubMed, SCOPUS, Science Direct, Cochrane Library, and Web of Science was conducted, following the Preferred Reporting Items for Systematic Reviews and Meta-Analyses 2020 guidelines to identify studies assessing IBS prevalence and risk factors in Saudi Arabia. A total of 22 studies involving 20,755 participants met the inclusion criteria. Most studies (21/22) used ROME IV criteria for IBS diagnosis, while one study used a self-administered questionnaire. The reported prevalence of IBS ranged from 2.7% to 83.3%, with an overall prevalence of 26.3% (5461 cases). Significant risk factors for IBS included depression, anxiety, a family history of IBS, female gender, student status, and middle age, while patients with IBS were more likely to exhibit higher levels of anxiety, depression, and nomophobia. Dietary habits also played a role, with the non-IBS group consuming more fiber and niacin, whereas the IBS group consumed more energy and carbohydrates. The findings of this systematic review highlight the substantial burden of IBS in Saudi Arabia and underscore the importance of dietary patterns, psychosocial factors, and genetic predisposition in its onset and severity. Given these insights, public health initiatives should focus on patient education and culturally tailored interventions for the effective management of IBS.

## Introduction and background

Irritable bowel syndrome (IBS) is a chronic functional gastrointestinal disorder characterized by altered bowel habits and abdominal pain in the absence of detectable structural abnormalities. The exact pathophysiology remains multifactorial, involving visceral hypersensitivity, dysregulation of the gut-brain axis, altered gut motility, and gut microbiome imbalances. Psychological stressors such as anxiety and depression, commonly observed in patients with IBS, have been linked to exacerbation of symptoms due to their effects on gut motility and hypersensitivity. 

According to the Rome IV criteria, IBS is classified into four subtypes based on predominant bowel habits. 1) IBS with predominant constipation (IBS-C): Hard or lumpy stools in >25% of bowel movements, with loose stools in <25%. 2) IBS with predominant diarrhea (IBS-D): Loose or watery stools in >25% of bowel movements, with hard stools in <25%. 3) IBS with mixed bowel habits (IBS-M): Both hard and loose stools occur in >25% of bowel movements. 4) Unclassified IBS (IBS-U): Bowel habit changes that do not fit into the above subtypes. Studies have identified IBS-D as the most common subtype in Saudi Arabia, particularly among young adults and medical students.

Diagnostic criteria

The diagnosis of IBS is primarily clinical and relies on the Rome IV criteria, which require recurrent abdominal pain at least one day per week in the last three months associated with at least two of the following: pain related to defecation, a change in stool frequency, and a change in stool form (appearance). These symptoms should have been present for the last three months, with onset at least six months prior to diagnosis [1].

 IBS prevalence and risk factors in Saudi Arabia

Globally, IBS remains a significant public health concern, with Saudi Arabia reporting high prevalence rates that impact quality of life, healthcare costs, and workforce productivity. Studies indicate IBS prevalence ranges from 10% to 20% and varies due to differences in diagnostic criteria, study populations, and regional influences. For instance, Almuzaini et al. [2] found a prevalence of 21.4% in the general population, while Alshaikh et al. [3] reported a 31.9% prevalence among university students. Similarly, a systematic review by Makkawy et al. [4] identified variations in IBS prevalence based on gender, lifestyle, and psychological factors. Notably, IBS is more prevalent in women than men, with a female-to-male ratio of approximately 2:1, a trend consistent with global research, attributing this difference to hormonal and psychosocial influences. Additionally, studies consistently report higher IBS prevalence among younger adults (18-35 years), particularly university students and young professionals, with contributing factors, including urbanization, dietary habits, and psychological stress. Research by Alshaikh et al. [3], Alhammadi et al. [5], and Mujamammi et al. [6] suggests that Westernized diets, processed food consumption, and low fiber intake exacerbate IBS symptoms. The increasing reliance on fast food, poor sleep patterns, and chronic stress due to modern lifestyles further elevate IBS risk [7,8].

Identifying the risk factors associated with IBS is crucial for both prevention and effective management. In the context of Saudi Arabia, several potential contributors have been recognized [9].

Firstly, dietary habits play a significant role in IBS development. In Saudi Arabia, the shift from traditional high-fiber diets to high-fat, processed foods has been linked to rapid urbanization and Westernized eating patterns, contributing to IBS symptom aggravation by altering gut microbiota and increasing gut hypersensitivity. Additionally, the high consumption of caffeinated and carbonated beverages, particularly among young adults and university students, has been associated with worsened IBS symptoms. Studies further suggest that these dietary shifts, combined with sedentary lifestyles and chronic stress, may exacerbate IBS prevalence in the region [10].

Secondly, psychological factors have a strong association with IBS. Conditions such as anxiety and depression can negatively impact gastrointestinal function, creating a bidirectional relationship where stress intensifies IBS symptoms and gastrointestinal discomfort, in turn, heightens stress levels. In Saudi culture, the stigma surrounding mental health issues may prevent individuals from seeking necessary help, which could contribute to the persistence of IBS symptoms Another contributor is the sedentary lifestyle that has become common, particularly among urban populations in Saudi Arabia. Physical inactivity can lead to irregular bowel movements and prolonged intestinal transit time, which increases the likelihood of developing IBS [1,7,11]. Furthermore, there is evidence of genetic predisposition playing a role in the development of IBS. A family history of gastrointestinal disorders may predispose certain individuals to similar conditions, suggesting a hereditary component in the manifestation of IBS symptoms [1,7,11].

Lastly, issues related to infections and dysbiosis can trigger the onset of IBS. Imbalances in the gut microbiome, along with previous gastrointestinal infections, have been identified as potential risk factors. Research has indicated that specific infections, such as those caused by enterotoxigenic *Escherichia coli*, can lead to post-infectious IBS, resulting in ongoing gastrointestinal distress [1,7,11].

IBS is a prevalent gastrointestinal disorder that significantly impacts individuals' quality of life. While global research on IBS is extensive, data specific to the Saudi population is limited. Understanding the epidemiology and risk factors of IBS within this population is crucial for effective prevention, management, and treatment strategies. The increasing prevalence of IBS worldwide, coupled with the lack of comprehensive data on the Saudi population, necessitates this systematic review.

Study objectives

The objectives of this study are to: (1) investigate the prevalence of IBS among different segments of the Saudi population (e.g., age, gender, geographic region); (2) examine the association between various demographic, lifestyle, and psychosocial factors and the development of IBS in the Saudi population; and (3) identify potential gaps in current knowledge regarding IBS in Saudi Arabia and suggest directions for future research.

## Review

Methods

This study adhered to the guidelines established by the Preferred Reporting Items for Systematic Reviews and Meta-Analyses [12] to conduct a systematic review examining the epidemiology and risk factors associated with IBS in the Saudi population. An electronic search was conducted across four databases such as PubMed, Web of Science, SCOPUS, and Science Direct to identify relevant studies published in English that investigate the prevalence and risk factors of IBS in this demographic. The search strategy incorporated keywords, such as "public health", "prevalence", "gastrointestinal disorders", "systematic review", "risk factors", "epidemiology", "Saudi Arabia", "IBS", "irritable bowel syndrome", related to IBS and its risk factors. Two reviewers independently screened the search results, selected eligible studies, extracted data, and evaluated the quality of the included research using appropriate assessment tools.

Inclusion Criteria

This systematic review considered studies that specifically focused on the Saudi population, encompassing both adults and children diagnosed with IBS. Eligible studies must be peer-reviewed original research articles, including various designs such as cross-sectional studies, cohort studies, and case-control studies that presented data regarding the prevalence and risk factors associated with IBS. Studies conducted in the last five years (2020-2024) were prioritized in this review. Additionally, only studies published in English were included. To ensure relevance and accuracy, the review considered research published within the last 15 years. The studies must specifically report on outcomes related to the epidemiology of IBS, including prevalence rates, demographic characteristics, and any associated comorbidities.

Exclusion Criteria

Conversely, the review excluded studies that did not concentrate specifically on the Saudi population or those that included mixed populations without providing specific data relevant to Saudi participants. Non-peer-reviewed articles, editorials, letters to the editor, opinion pieces, and conference abstracts were also disregarded. Additionally, studies published in languages other than English were not included. Research that does not address IBS or its associated risk factors, such as investigations solely focused on other gastrointestinal disorders, was excluded. Lastly, studies that lack sufficient data on the prevalence or risk factors of IBS were omitted to ensure that only meaningful analyses were incorporated into the review. These criteria facilitated the selection of high-quality and relevant studies that enhanced the understanding of the epidemiology and risk factors of IBS within the Saudi population.

Data Extraction

To ensure precision, the search results were verified using Rayyan (Rayyan Systems Inc., Cambridge, MA, USA) [13]. Titles and abstracts retrieved in the search were evaluated for relevance according to the inclusion and exclusion criteria. Papers meeting the inclusion criteria underwent detailed review by the research team. Any discrepancies were resolved through consensus. Key study information, including titles, authors, publication year, study location, participant demographics, gender distribution, and epidemiology and risk factors of IBS in Saudi Arabia, were recorded using a predefined data extraction form. An independent assessment tool was developed to assess the risk of bias.

Data Synthesis Strategy

In order to provide a qualitative evaluation of the research findings and components, summary tables were generated using data extracted from relevant studies. Once the data collection for the systematic review was completed, the optimal approach for using the data from the included studies was determined.

Risk of Bias Assessment

For evaluating the study's quality, we employed the Joanna Briggs Institute (JBI) critical assessment criteria for prevalence studies [14]. This tool consists of nine questions, each designed to assess potential sources of bias and the overall reliability of prevalence data. Each question is rated as 1 (yes) for a positive response and 0 (no, unclear, or not applicable) for a negative or ambiguous response. The total scores classify studies into low (≤4), moderate (5-7), and high (≥8) quality categories. Any discrepancies in assessment were resolved through discussion among researchers. The nine questions in the JBI checklist assess key methodological aspects, including whether the sample frame was appropriate to address the target population, ensuring that the study population represents the broader target population without selection bias. The tool also evaluates whether study participants were sampled appropriately using methods such as random, systematic, or stratified sampling to minimize bias. It further assesses whether the sample size was adequate to provide reliable prevalence estimates and if the study subjects and setting were described in detail, ensuring transparency regarding participants and study locations. The checklist considers whether data analysis covered the identified sample comprehensively, if valid diagnostic methods (e.g., Rome IV criteria for IBS) were applied, and whether the condition was measured in a standard, reliable way across all participants. Additionally, it evaluates the appropriateness of statistical analysis and the adequacy of the response rate, ensuring that any low response rate was managed appropriately to minimize potential bias. By including these details, we aim to enhance transparency regarding how risk of bias was assessed and categorized in our systematic review.

Results

Systematic Search Outcomes

**Figure 1:**
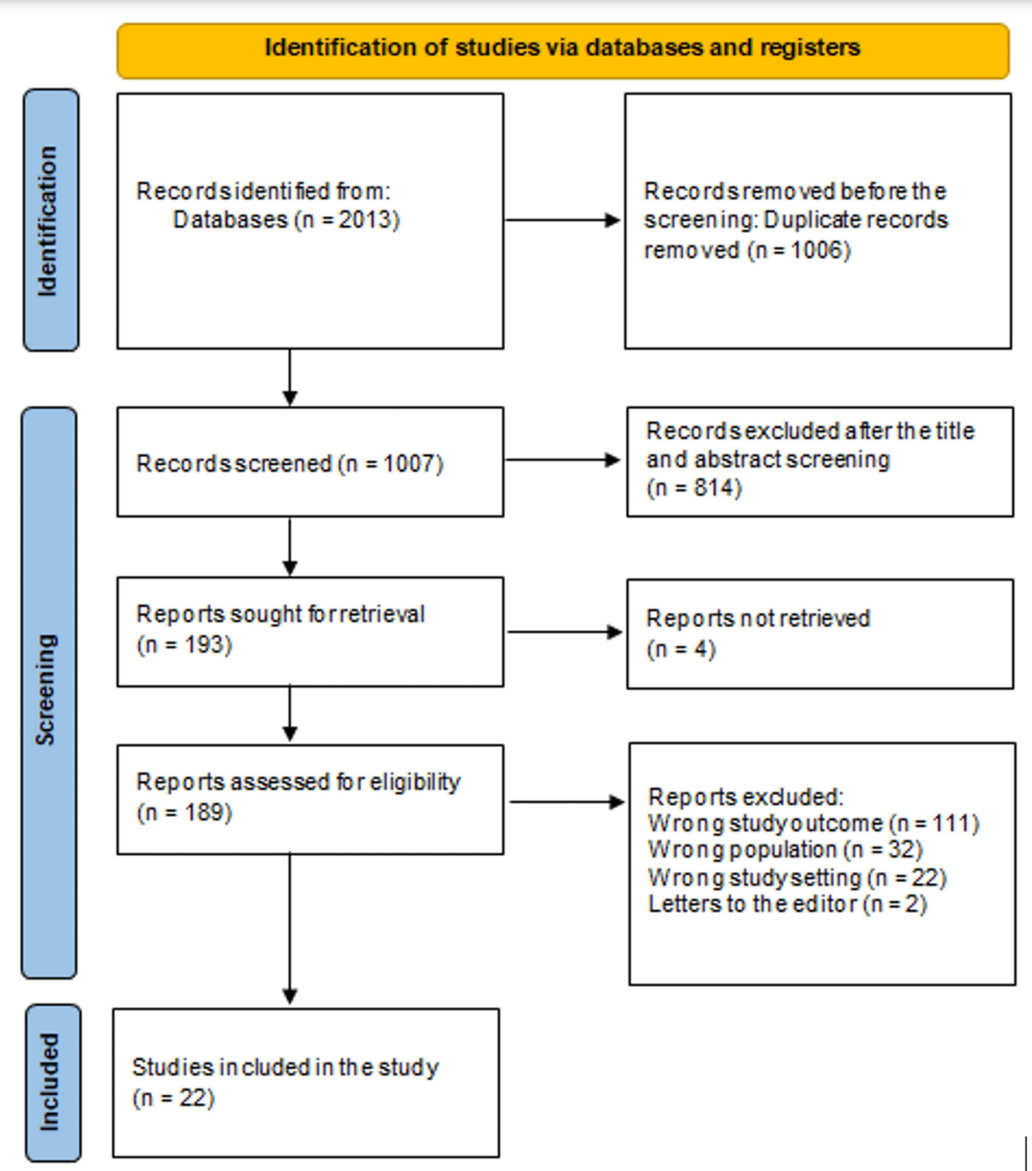
Summarizes the study decisions using a PRISMA diagram PRISMA: Preferred Reporting Items for Systematic Reviews and Meta-Analyses

Around 2013 studies were identified from the database searches. After removing 1006 duplicates, 1007 research papers remained. Upon examining the abstracts and titles of 1007 investigations, 814 papers were rejected. Four articles were missing from the 193 reports that were required. 189 articles made it through the full-text screening process; however, 32 were rejected due to inappropriate population type, 2 were editor's letters, 22 were excluded due to an irrelevant study setting, and 111 were rejected because their study results were irrelevant. Twenty-two research publications that were included in this systematic review met the eligibility criteria. A diagram of the methodology used to choose the literature is shown in Figure 1. 

*Sociodemographic Characteristics* *of the Comprised Participants and Studies*

The sociodemographic information obtained from the research publications is shown in Table 1. Our data included 22 trials with 20,755 participants, of whom almost half, 10,371 (49.9%), were males. All of the included articles were cross-sectional studies [2,3,6,9,10,15-30]. The clinical data are presented in Table 2. Twenty-one out of the 22 included studies used ROME IV criteria for IBS diagnosis, and only one used a self-administrated questionnaire [16]. Only seven studies included university students [3,6,18,23,24,28,30], one included physicians [29], and 12 included the Saudi general population [5,10,2,15-17,19,9,20-22,25-27]. The prevalence of IBS ranged from 2.7% [18] to 83.3% [16], with a total prevalence of 26.3% (5461). Depression [3], anxiety [3,17], a family history of IBS [2,3,17], female gender [2,5,6,10,19], single status [17], and middle age [5] were found to be the main risk factors for IBS in students. Individuals with IBS frequently experience higher levels of anxiety, depression, migraines, and nomophobia [3,15,16,18]. While the non-IBS group consumed much more fiber and niacin, the IBS group consumed significantly more energy, carbohydrates, and saturated fatty acid (SFA) [6]. A significant familial predisposition to IBS is consistently observed, with family history playing a prominent role. Psychological stress, including anxiety and depression, emerges as a major contributor, particularly among students and individuals facing challenging work or educational environments. In school and university contexts, IBS prevalence was heightened by academic stress, irregular routines, and poor sleep patterns. Among physicians and medical students, extended work hours and chronic stress were significant risk factors [3,6,18,23,24,28,29,30].

**Table 1:** Sociodemographic parameters of the involved populations NM: Not mentioned

Study	Study design	City	Participants	Mean age	Males (%)
Alhazmi et al., 2024 [10]	Cross-sectional	Jazan	637	31.2 ± 10.5	307 (51.8%)
Alshaikh et al., 2024 [3]	Cross-sectional	Abha	379	21.7 ± 2.9	202 (53.3%)
Almuzaini et al., 2024 [2]	Cross-sectional	Qassim	402	18 to >50	74 (18.4%)
Sheikh et al., 2024 [15]	Cross-sectional	Makkah & Al-Madinah	1022	18 to >60	256 (25%)
Aljahdli et al., 2024 [16]	Cross-sectional	Jeddah	1346	18-70	364 (27%)
Alkalash et al., 2023 [17]	Cross-sectional	Al-Qunfudah	355	28.7 ± 8	147 (41.4%)
Alhammadi et al., 2023 [5]	Cross-sectional	Asser	683	20 to >60	99 (14.5%)
Agwa et al., 2023 [18]	Cross-sectional	Al-Baha	452	21.6 ± 1.8	285 (63.1%)
Hafiz et al., 2023 [19]	Cross-sectional	Makkah	936	25-55	435 (46.5%)
Mujamammi et al., 2023 [6]	Cross-sectional	Riyadh	426	21.2 ± 1.6	271 (63.1%)
Alshahrani et al., 2023 [20]	Cross-sectional	Southern Saudi Arabia	1622	NM	900 (55.5%)
Alqahtani et al., 2022 [9]	Cross-sectional	Taif	1680	32.2 ± 12.3	604 (36%)
Alharbi et al., 2022 [21]	Cross-sectional	Makkah	921	18->60	374 (40.6%)
Basharat et al., 2022 [22]	Cross-sectional	Aseer	6300	NM	4200 (66.7%)
Fadl et al., 2022 [23]	Cross-sectional	Multi-centered	300	NM	109 (36.3%)
El-Gamal et al., 2022 [24]	Cross-sectional	Jeddah	346	23.2 ± 2.6	171 (49.4%)
Arishi et al., 2021 [25]	Cross-sectional	Jazan	1554	18-69	860 (55.3%)
Amin et al., 2021 [26]	Cross-sectional	Riyadh	1319	18->60	706 (53.5%)
Alanazi et al., 2021 [27]	Cross-sectional	Arar	230	16.9 ± 0.8	0
AlButaysh et al., 2020 [28]	Cross-sectional	Medina	767	21.9 ± 1.9	337 (43.9%)
AlAmeel et al., 2020 [29]	Cross-sectional	Dammam	549	47	419 (76.3%)
Alzahrani et al., 2020 [30]	Cross-sectional	Majmah	151	22-25	151 (100%)

**Table 2:** Clinical parameters and outcomes of the comprised research FODMAP: Fermentable oligosaccharides, disaccharides, monosaccharides, and polyols; GAD: Generalized anxiety disorder; GERD: Gastroesophageal reflux disease; IBS: Irritable bowel syndrome; IBS-M: IBS with mixed bowel habits; JBI: Joanna Briggs Institute; NM: Not mentioned; NSAID: Nonsteroidal anti-inflammatory drug; SFA: Saturated fatty acid

Study ID	IBS diagnostic tool	Population type	Prevalence of IBS (%)	Main outcomes	JBI
Alhazmi et al., 2024 [10]	ROME IV	General population	198 (31.08%)	It was found that women experienced IBS at a considerably higher rate than men.	Moderate
Alshaikh et al., 2024 [3]	ROME IV	University students	121 (31.9%)	IBS is remarkably common among college students, particularly among those pursuing non-health-related specializations. Individuals with IBS frequently experience higher levels of anxiety and despair; nonetheless, the poorer levels of well-being among IBS students were not statistically significant. Depression, anxiety, and a family history of IBS were found to be the main risk factors for IBS in students.	Moderate
Almuzaini et al., 2024 [2]	ROME IV	General population	86 (21.4%)	There was a strong positive correlation—especially in females—between food allergies and IBS. A major predictor of IBS was a family history, with men being more susceptible.	Moderate
Sheikh et al., 2024 [15]	Questionnaire	General population	153 (15%)	Individuals with probable depression and IBS and those who used mobile devices more frequently had a significantly increased risk of developing nomophobia.	High
Aljahdli et al., 2024 [16]	ROME IV	General population	1,121 (83.3%)	The majority of patients had IBS-M, and 38.1% of participants said that their gastrointestinal symptoms were extremely bothersome. Additionally, 47.1% of patients reported that their symptoms often or continuously interfered with their everyday activities. Though 20.4% and 19.8% of patients felt angry or depressed, respectively, 25.0% of patients tolerated these bothersome symptoms.	High
Alkalash et al., 2023 [17]	ROME IV	General population	102 (30.4%)	IBS risk variables included being single, having a positive family history of the condition, and having GAD. People must be made aware of the signs and effects of IBS.	Moderate
Alhammadi et al., 2023 [5]	ROME IV	General population	273 (39.9%)	The outcomes highlight a strong relationship between these two circumstances. Many risk factors have been identified, including middle age, female gender, migraine history in the family, and mental health issues, all of which increase susceptibility to these illnesses.	Moderate
Agwa et al., 2023 [18]	ROME IV	University students	12 (2.7%)	This hypothesis is contradicted by the lack of substantial differences in prevalence between participants who are medical students and those who are not. The study also demonstrates the importance of lifestyle choices such as exercise in the management and avoidance of migraines and IBS in medical students.	Moderate
Hafiz et al., 2023 [19]	ROME IV	General population	420 (44.9%)	In the majority of the studies, patients with IBS were married women in their 25s to 35s with IBS-M. IBS has been linked to age, gender, marital status, and occupation.	Moderate
Mujamammi et al., 2023 [6]	ROME IV	University students	76 (17.8%)	IBS affects 17.8% of medical students, with a higher frequency in females. While the non-IBS group consumed much more fiber and niacin, the IBS group consumed significantly more energy, carbohydrates, and SFA. The consumption of FODMAPs and IBS did not significantly correlate according to our findings.	Moderate
Alshahrani et al., 2023 [20]	ROME IV	General population	700 (43.2%)	When comparing gender and IBS prevalence, we found no discernible differences.	Moderate
Alqahtani et al., 2022 [9]	ROME IV	General population	306 (18.2%)	A positive family history of IBS is the most prevalent risk factor among patients with IBS (80%).	
Alharbi et al., 2022 [21]	ROME IV	General population	186 (20.2%)	Stress-diagnosed patients were 2.5 times more likely to have IBS than an intact person, and participants with anxiety and depression in the current study were statistically significantly more likely to have IBS than those without these conditions.	Low
Basharat et al., 2022 [22]	ROME IV	General population	1500 (23.8%)	It has been shown that smoking habits, GERD, food sensitivity, anxiety, mental stress, a family history of IBS, the usual use of NSAIDs, and the disease prior to the occurrence of side effects are the main causes of IBS in Saudi Arabia.	Low
Fadl et al., 2022 [23]	ROME IV	University students	148 (49.3%)	IBS was predicted with female gender, better academic standing, less activity, and sleep disturbance.	Moderate
El-Gamal et al., 2022 [24]	ROME IV	Medical students	60 (17.3%)	Students attending private colleges were more likely to have it. Anxiety and smoking were identified as key factors that contribute to IBS.	Moderate
Arishi et al., 2021 [25]	ROME IV	General population	248 (16%)	IBS was substantially linked to mental health conditions, including stress and anxiety, female sex, and tobacco use.	Moderate
Amin et al., 2021 [26]	ROME IV	General population	104 (7.9)	IBS is most strongly associated with job status, low family income, and female gender.	Moderate
Alanazi et al., 2021 [27]	ROME IV	School students	126 (15.5)	IBS was substantially correlated with school grade, marital status, low family income, having more children, and parent status (p<0.001).	Moderate
AlButaysh et al., 2020 [28]	ROME IV	University students	121 (15.8%)	Female sex, attending a medical school, renting an apartment, living on campus, having a family history of IBS, smoking, exercising, having poor sleep, and experiencing mental stress were all risk factors for IBS.	Moderate
AlAmeel et al., 2020 [29]	ROME IV	Physicians	89 (16.3%)	IBS was more prevalent among younger doctors, men, and those who put in more hours at work.	Moderate
Alzahrani et al., 2020 [30]	ROME IV	Medical students	11 (7.3%)	Students with anxiety, a history of chronic health issues, mental stress, and food sensitivity were more likely to have IBS.	Low

Discussion

Dietary habits in Saudi Arabia, marked by a high intake of fat, spicy foods, and low fiber, significantly contribute to IBS symptoms. Traditional meal patterns, such as large evening meals and frequent consumption of rich dishes during social gatherings, may exacerbate symptoms. Additionally, the cultural emphasis on hospitality and shared meals often leads to excessive consumption of high-calorie foods, which can impact gut health. Moreover, these cultural factors further highlight the importance of targeted public health interventions tailored to the Saudi population. This problem is made worse by the rising tendency of fast-food consumption, especially among younger people. The research also emphasizes the significance of psychological elements such as stress and worry, which are common in Saudi society and have a strong correlation with symptoms of IBS [9]. This review found that the prevalence of IBS in Saudi Arabia ranged from 2.7% [18] to 83.3% [16], reflecting substantial variability across studies. This wide range may be attributed to differences in study populations, with some studies focusing on university students, physicians, or the general population. Additionally, variations in diagnostic methods (e.g., Rome IV criteria vs. self-reported questionnaires), sample sizes, and geographic distribution across urban and rural areas may have influenced the reported prevalence. The highest reported prevalence (83.3%) was found in a study focusing on individuals with severe gastrointestinal symptoms, whereas the lowest prevalence (2.7%) was observed in a cohort with different lifestyle and dietary patterns. These disparities highlight the need for standardized diagnostic approaches and larger, more representative population studies to establish more precise IBS prevalence estimates in Saudi Arabia, with a total prevalence of 5461 (26.3%). This was higher than a similar Saudi review (24%) by Makkawy et al. [4] and (20.7%) Almasary et al. [31]. According to a study by Alosaimi et al. [32], the prevalence of IBS varied between 8.9% and 31.8% in Arab nations. According to a different comprehensive study that was published in 2020, the prevalence of IBS worldwide, based on Rome III or Rome IV criteria, was 3.8% and 9.2%, respectively, with a predominance of women across 38 nations [33]. Given that the prevalence varies geographically, our results were similar to the global prevalence.

We found that depression [3], anxiety [3,5], a family history of IBS [2,3,5], female gender [2,5,6,18,20], being single [5], and middle age [18] were the main risk factors for IBS in students. While the non-IBS group consumed much more fiber and niacin, the IBS group consumed significantly more energy, carbohydrates, and SFA [20]. Similarly, Makkawy et al. reported that IBS has been found to be influenced by a variety of factors in different cohorts, including living situations, mental health, dietary habits, family history of the disorder, and some comorbidities, including diabetes mellitus. In addition, lifestyle variables such as dehydration, poor dietary fiber, stress, and even caffeine consumption were linked to IBS [4].

In addition to dietary habits, psychological stressors, and genetic predisposition, sociocultural factors may play a crucial role in the prevalence and severity of IBS in Saudi Arabia. The stigma surrounding mental health conditions, which have been widely recognized in Saudi society, may prevent individuals from seeking timely psychological or medical interventions, thereby exacerbating IBS symptoms. Symptoms are often underreported due to cultural taboos associated with mental health discussions [3,18]. This reluctance may contribute to the persistence of symptoms and a higher burden of undiagnosed cases within the population. Hormonal fluctuations are thought to affect gut sensitivity and function, particularly in women, which could account for the gender gap. Another aspect is age, with some age groups exhibiting a higher prevalence than others, such as the 51-60 age group [34]. There could be a variety of contributing factors, such as accumulated life stressors, dietary modifications, or age-related physiological changes.

According to a survey conducted on Egyptian medical students, the prevalence of IBS ranged from 9.3% to 35.5%. Stressful environments were the main cause of the high incidence among students, even in the case of female gender, family history, psychiatric disorders, depression, anxiety, infections, food habits, and sleep patterns and quality [35]. Based on 22 cross-sectional studies, a systematic review and meta-analysis of Chinese university students found that the pooled prevalence of IBS was 11.89%. In this study, smoking, drinking, anxiety, and depression were the most related factors [36].

To gain a deeper understanding of the natural history of IBS in the Saudi population and to spot any possible variations in prevalence over time, future research should concentrate on longitudinal studies. Research on the genetic variables specific to the Saudi population that could contribute to the development of IBS is required. Furthermore, studies should investigate the effectiveness of culturally specific interventions such as dietary changes, psychological counseling, and patient education initiatives in managing IBS.

Given that IBS is often exacerbated by irregular sleep patterns and heightened stress levels [24,34], investigating its prevalence among healthcare workers, shift employees, and professionals in high-pressure jobs could provide a more comprehensive understanding of occupational influences on IBS [30,37].

There are various restrictions on this review. Firstly, there is limited literature on IBS prevalence across all population groups of Saudi Arabia. Additionally, there is a lack of studies on IBS in rural populations, as most research focuses on urban settings.

There is variability in demographic samples and diagnostic criteria among the included studies. Secondly, the reliance on self-reported data poses a potential risk of recall bias or underreporting, particularly in cultural settings where gastrointestinal symptoms may be stigmatized.

Thirdly, the inclusion of studies employing different methodologies could lead to inconsistencies in the reported prevalence and risk factors of IBS, making it challenging to synthesize findings across studies. This variability may impact the generalizability of the results.

Finally, there is a need for further research to address existing knowledge gaps on IBS in Saudi Arabia. Future studies should aim to include diverse population groups, use standardized diagnostic criteria, and examine both urban and rural populations to enhance the accuracy and applicability of findings.

## Conclusions

This comprehensive review found a high prevalence of IBS in the Saudi population. The study also highlights the important roles that dietary practices, psychosocial variables, and genetic predisposition play in the onset and severity of the illness. The results emphasize the necessity of patient education and culturally competent public health measures for the successful management of IBS.
